# Assembly of Graphene Platelets for Bioinspired, Stimuli-Responsive, Low Ice Adhesion Surfaces

**DOI:** 10.1021/acsomega.1c06782

**Published:** 2022-03-17

**Authors:** Yuequn Fu, Senbo Xiao, Bjørn Helge Skallerud, Zhiliang Zhang, Jianying He

**Affiliations:** NTNU Nanomechanical Lab, Department of Structural Engineering, Norwegian University of Science and Technology (NTNU), Trondheim 7491, Norway

## Abstract

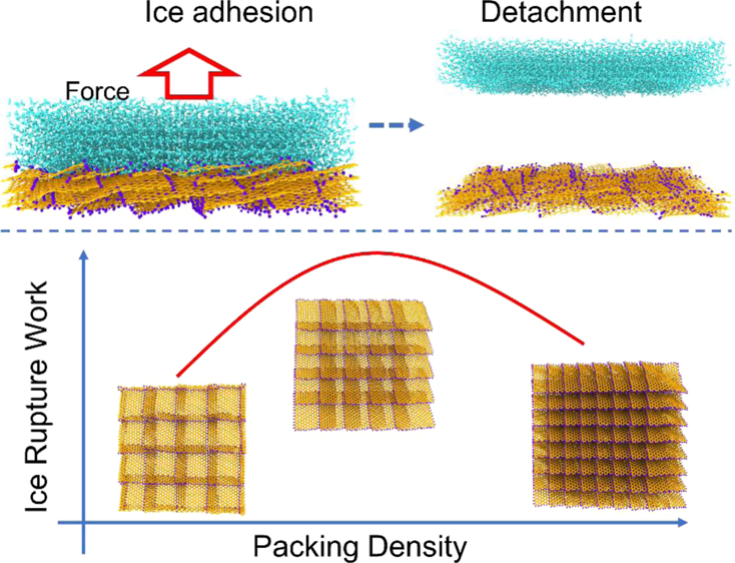

Design and fabrication of functional materials for anti-icing and deicing attract great attention from both the academic research and industry. Among them, the study of fish-scale-like materials has proved that enabling sequential rupture is an effective approach for weakening the intrinsic interface adhesion. Here, graphene platelets were utilized to construct fish-scale-like surfaces for easy ice detachment. Using a biomimicking arrangement of the graphene platelets, the surfaces were able to alter their structural morphology for the sequential rupture in response to external forces. With different packing densities of graphene platelets, all the surfaces showed universally at least 50% reduction in atomistic tensile ice adhesion strength. Because of the effect of sequential rupture, stronger ice–surface interactions did not lead to an obvious increase in ice adhesion. Interestingly, the high packing density of graphene platelets resulted in stable and reversible surface morphology in cyclic tensile and shearing tests, and subsequently high reproducibility of the sequential rupture mode. The fish-scale-like surfaces built and tested, together with the nanoscale deicing results, provided a close view of ice adhesion mechanics, which can promote future bioinspired, stress-responsive, anti-icing surface designs.

## 1 Introduction

**Figure 1:**
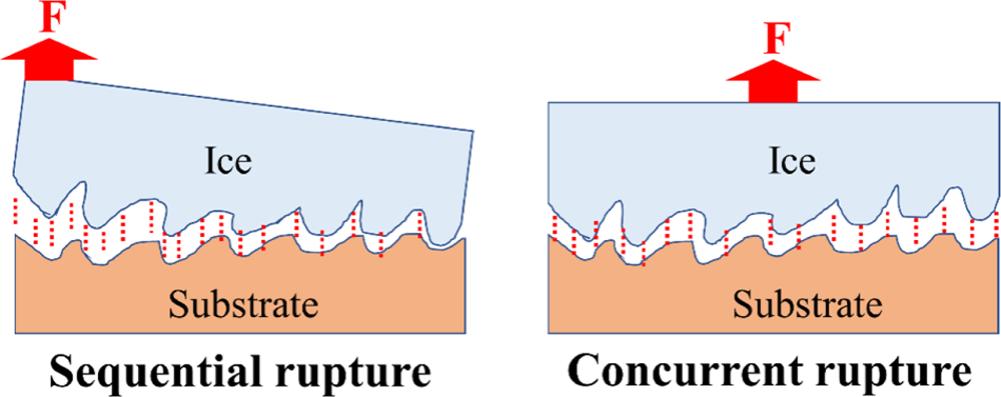
Sequential and concurrent rupture between the ice and its adhering substrate. Atomistic interactions, indicated as red dashed lines, are broken in an incremental manner in sequential rupture (left panel), while all at once in the concurrent rupture mode (right panel). Given the same number and strength of atomistic interactions, the sequential rupture mode leads to a lower rupturing force.

Unwanted icing is one of the major challenges to infrastructure and human activities in environments below water freezing temperature.^1,2^ For instance, atmospheric icing including precipitation, in-cloud, and frost, directly results in problems of electrical failure, overproduction, power losses, measurement errors, and safety hazards on wind turbines^3,4^ at high altitudes. Ice accretion is also a lethal hazard to aircraft^5,6^ due to its icing effects on the handling and performance of the wings. Icing combined with wind could cause damage and power outages on power networks. Highly relevant to our daily life during winter or in cold regions, icing could blot out the visual field from the windshield, causing inconvenience to drivers or passengers. Many applications of anti-icing or deicing have been used to prevent or minimize icing effects, aiming at lifetime extension, energy saving, and cost reduction.^4,5,7−12^ Materials with super-low ice adhesion strength are highly desired in addressing the icing problem and are under active development today.^13−15^ After identifying the determinants of ice adhesion, it is recognized that intrinsic ice adhesion is a key factor for the firm attachment of ice on different surfaces.^1,16,17^ Specifically, for a seemingly ice-covered area on a rough surface on the macroscale, termed apparent adhesion, only the truly effective contacting points or areas and interlockings on the nanoscale, termed intrinsic adhesion, are responsible for the observed ice adhesion strength.^1^ Seeking low intrinsic ice adhesion strength can rely not only on physical chemistry level atomistic interactions,^8^ for instance, using superhydrophobic materials, but also on the design of the stress-responsive rupture mode of atomistic ice–substrate interactions.^19,20^ Considering the full detachment of an intrinsic contacting area as depicted in Figure 1, the sequential rupture between the ice and its substrate leads to much lower rupture force, and thus stress, than the concurrent breakage of all the atomistic interactions at once.^19^

Inspiring by the application of biomaterials^21,22^ to designing materials with low intrinsic ice adhesion, natural surfaces can provide inspiring guidelines. There are many natural surfaces with topography for tailored mechanical functions, especially the ones that are able to respond to external stress in wetting and adhesion.^23^ Two outstanding examples in this regard are the hierarchical surfaces of water strider legs and gecko toes.^24,25^ These two nature-designed surfaces consist of hierarchical structures of small, flexible, and organized units for realizing tailored properties. Specifically, the uniquely oriented needle-like microsetae on water strider legs enable superior water repellence, and well-organized setae on gecko toes enable fast switching between strong attachment and easy detachment. The microsetae on water strider legs are superhydrophobic, namely having super-low adhesion to water.^26^ In comparison, the setae on gecko toes can on the one hand, apply strong van der Waals forces on different surfaces,^27,28^ and, on the other hand, can easily detach from solid surfaces by the rolling of the gecko feet, namely by sequential rupture of the setae–substrate interactions.^25,29^ The ordered oriented microsetae and setae on the two surfaces are made for sequential rupture of any adhesion, which is demonstrated in the detachment process of gecko toes from different surfaces. Most importantly, the weak adhesion of gecko toes to solid substrates is highly reusable, which is enabled by the optimized packing of the setae in the surface hierarchical structures. Mimicking the organization and the mechanical functions of such natural surfaces by featuring their surface topography can serve as a practical approach for lowering intrinsic ice adhesion.^19^

**Figure 2:**
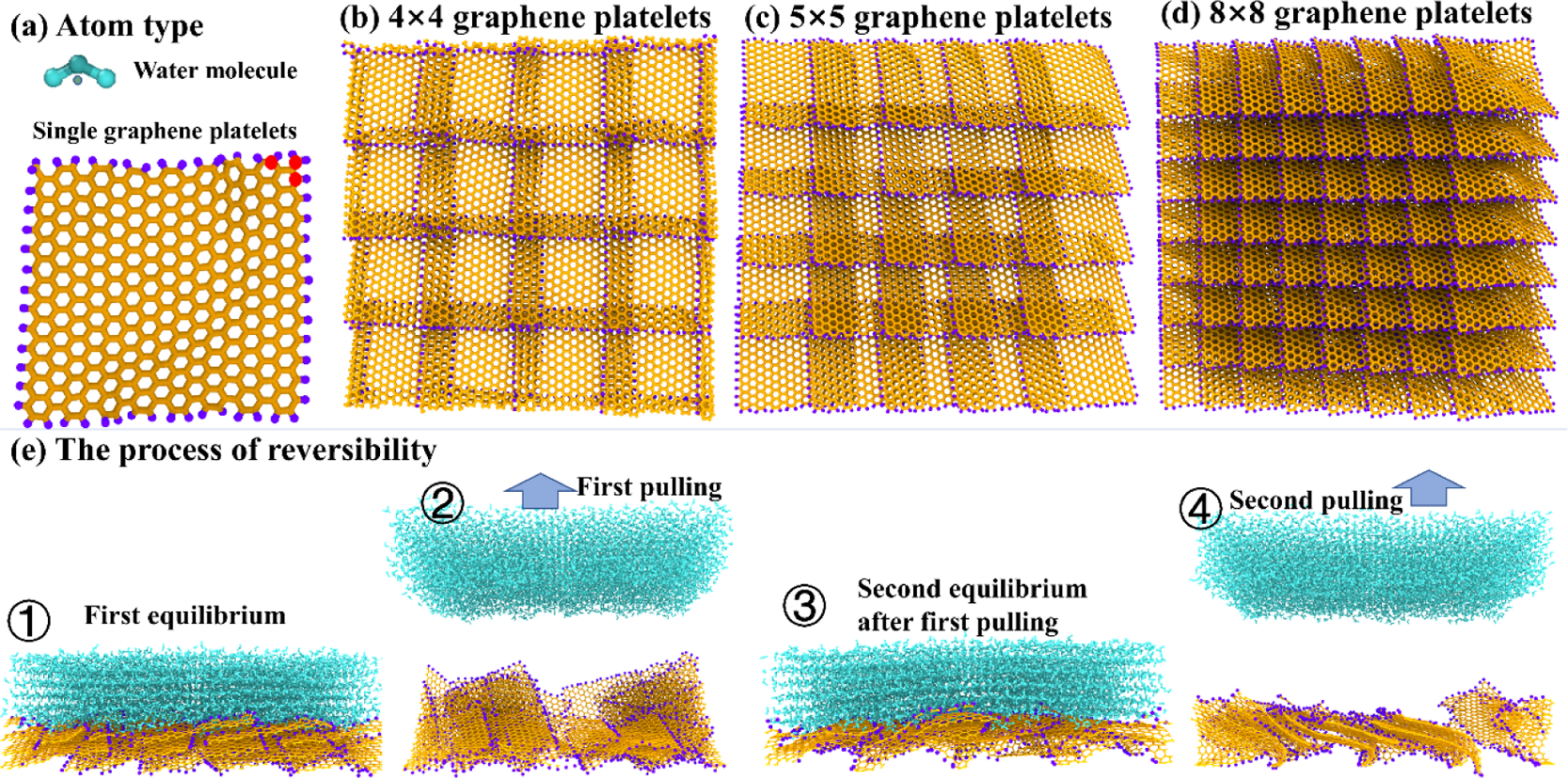
Atomistic models and cyclic deicing. (a) Tip4p/ice water model and the graphene platelet. Atoms on the graphene platelets that are fixed to enable sequential rupture are highlighted in red. (b–d) C4, C5, and C8 fish-scale-like surfaces from left to right, with a low to high graphene packing density. (e) Adopted cyclic deicing procedure, including ice equilibration adhesion on the surfaces, first round detachment, re-adhesion, and re-detachment.

Former studies have illustrated that sequential rupturing of atomistic interactions can lead to weaker adhesion, which was also applied to low intrinsic ice adhesion strength.^19^ Using graphene platelets for constructing a fish-scale-like surface as shown in Figure 2a,b, the previous study realized the two rupture modes of sequential and concurrent ice detachment from its adhering substrates. Strikingly, the sequential rupture mode of ice detaching can lead to a ∼60% reduction in ice adhesion strength.

Given the role of the sequential rupture mode in lowering ice adhesion, an interesting question that awaits an answer is how the arrangement or packing of graphene platelets in the fish-scale-like surface affects the intrinsic ice adhesion in attaching-and-detaching cycles. Addressing this question can further verify the fish-scale-like surface for successful anti-icing and guide the pattern design. As such, this work modeled and systematically compared ice adhesion and friction on fish-scale-like surfaces with varied packing densities of graphene platelets. This study aims at icephobic surface design for low intrinsic ice adhesion, and it also serves as a reference for the nanoscale interface tribology of snow and ice on solid surfaces.

## 2 Computational Details

The focus of atomistic modeling is to construct fish-scale-like surfaces with a low to high density of graphene platelets. The resulting surfaces are subjected to molecular dynamics (MD) simulations to probe the ice adhesion strength and the morphological evolution of the hierarchical surface structure after repeating icing and deicing cycles.

### 2.1 Modeling

Following the modeling procedure in the previous study,^19^ graphene platelets of the same size and geometry were used in constructing the fish-scale-like surfaces here, as shown in Figure 2a. The graphene platelets have a uniform area of 2.3 × 2.3 nm^2^. In an area of 10.4 × 10.4 nm^2^ on the *XZ*-plane, the graphene platelets were arranged to feature fish scales of different coverage densities as shown in Figure 2b–d. Specifically, the coverage densities included marginal edge covering of 4 × 4 graphene platelets in the *X*- and *Z*-direction (Figure 2b, termed “C4”), a medium covering density of 5 × 5 graphene platelets (Figure 2c, termed “C5”), and a close packing of 8 × 8 graphene platelets (Figure 2d, termed “C8”). It should be noted that the C4 model was identical to the fish-scale-like surface reported in the previous study.^19^ With the periodicity of the simulation box, the resulting three fish-scale-like surfaces were also periodic in the *XZ*-plane. All the graphene platelets on the three surfaces followed the same tilting orientation along the *X*- and *Z*-axis. As such, the sequential rupture mode was enabled if all the graphene platelets of the three surfaces were fixed at a unique corner (labeled by red atoms), as shown in Figure 2a. In contrast, the concurrent rupture was enabled if the whole graphene platelets were fixed.

The most commonly observed ice in the biosphere, hexagonal ice (*I*_h_),^30^ was used for testing ice adhesion on the three fish-scale-like surfaces. Same as in the previous study,^19^ a periodic ice layer on the *XZ*-plane with a thickness of ∼2 nm was placed onto each fish-scale-like surface, with the basal face (0 0 0 1) of the ice facing the surfaces. The ice and the surfaces had no atomic overlap initially in order to avoid high energy spots disturbing the stability of the system. At the same time, the ice and the surfaces were adjacent in the interaction cutoff distance, enabling a fast adhesion process in the following MD simulations.

The detailed atomic parameters used in this work are kept the same as in the previous studies of nanoscale deicing.^19,31^ The tip4p/ice was chosen to model the ice structure and the OPLS force-field was chosen for the graphene platelets.^32,33^ Such a model is well suited to performing coexistence points under different ice configurations and liquid water by using a generalized Gibbs–Duhem integration.^34^ The parameters of fusion carbon number 9 in naphthalene and a hydrogen atom from benzene were adopted for the carbon and hydrogen atoms in the graphene platelets, respectively.^19,33^ All the graphene platelets were electrically neutral and interacted with the ice via the Lennard-Jones potential. A nonbonded interaction cutoff distance of 1.0 nm was chosen for all the systems.

### 2.2 Simulations

All the MD simulations were performed using package GROMACS 5.0.7.^35^ Simulation boxes with periodic boundary conditions were used for all the systems. As all the fish-scale-like surfaces were periodic in the *XZ*-plane, the simulation boxes with periodic boundary conditions had the same *XZ* dimension as the surfaces. All the simulation boxes held 20 nm of buffer space on the *Y*-axis, more than two times longer than the combined thickness of the ice and the fish-scale-like surfaces, which guaranteed no interactions of the systems with their virtual images on the *Y*-axis of the simulation boxes.

The atomistic structures of the systems were first energy minimized using the steepest descent algorithm before carrying out ice adhesion and deicing simulations. All the MD simulations were carried out in the canonical *NVT* ensemble (a canonical ensemble is defined by these three parameters: the number of particles *N* in the system, the system’s volume *V*, and the system’s temperature *T*, each of which can have an effect on the system’s internal states), with a simulation time step of 2 fs. In order to maintain the stable ice structure, a temperature of 180 K was chosen for all the systems, as in the previous studies.^36,37^ The Nosé–Hoover coupling method with a coupling time of 0.4 ps was used to control the temperature of the simulation systems.^38,39^ Equilibration simulations with a length of 100 ns were then performed for the ice to adhere onto the three fish-scale-like surfaces and for the graphene platelets to adjust their position on the surfaces upon ice adhering. The final system snapshots of the equilibration simulations were then taken for nanoscale cyclic deicing tests of pulling and shearing.

Nanoscale cyclic tests were conducted by first detaching fully equilibrated ice adhered to the fish-scale-like surfaces, and then letting the ice re-adhere back onto the surfaces for 100 ns of equilibration again, and finally detaching once more. As shown in Figure 2e, the morphology evolution of the fish-scale-like surfaces and the resulting ice adhesion strength can be compared. Given the high computational cost, two rounds of ice adhesion and deicing were carried out to test the reversibility of the morphologies of the fish-scale-like surfaces. The purpose was thus limited to the comparison among the three surfaces in realizing the sequential rupture. To apply tensile and shearing forces to the ice, a virtual harmonic spring with an elastic force constant of 2000 kJ/mol/nm^2^ was tethered to the center of the mass (COM) of the ice, similar to the previous study.^20,40^ For generating tensile force, the spring was set to move at a constant speed of 0.5 nm/ns along with the *Y*-axis direction, also vertically away from the fish-scale-like surfaces. To enable shearing, the moving direction of the spring was set to be along or against the *Z*-axis direction. The tensile and shearing forces were then generated with the increasing distance between the COM of the ice and the spring position, which was recorded every 5 ps. The ice adhering tensile stress (σ) was calculated using the tensile force normalized by the cross-sectional area of the simulation box on the *XZ*-plane (A), namely the apparent area of the surfaces, as shown in eq 1. The tensile rupture stress (σ) denotes the peak value of the tensile ice detachment stress. The shearing stress (τ) was calculated using the monitored shearing force divided by the apparent area of the surfaces (*A*), as shown in eq 2. For statistical significance, five independent simulations were carried out for each of the nanoscale deicing tests. The rupture work needed to detach the ice from the fish-scale-like surfaces was calculated by integrating the pulling force along with the separation distance between the ice and the surfaces. Given that the loading rate can affect σ,^19^ two pulling speeds of 0.2 and 1 nm/ns were also used to probe the deviation in the results. As a continuous work of the previous study,^20^ this work aims to investigate the reversibility of these fish-scale-like materials and explore the package density’s effect on the ice adhesion, which is expected to supply the guidelines for anti-icing or deicing materials’ design and fabrication.
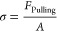
1
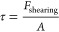
2

## 3 Results and Discussion

### 3.1 Cyclic Tensile Deicing on the Fish-Scale-Like Surfaces

The most interesting mechanical properties of the fish-scale-like surfaces are their ability to enable the sequential rupture for the purpose of weakening adhesion strength.^19^ Because of the different packing densities of the graphene platelets, the surface area contacting the ice in the three systems varied, as shown in Figure S1. Specifically, the C4 and C5 surfaces with a low packing density showed a larger surface area than the C8 surface with a high packing density, as shown by Figure 2b–d and Supporting Information Figure S2. Correspondingly, the equilibrated ice adhering interfaces also differed on the three surfaces, namely the large rough ridges observed on the equilibrated ice interface on C4 and C5 but small tips on C8, as shown in Supporting Information Figure S2. By comparing the interaction potential between the ice and the three surfaces, the C5 surface had the strongest interaction thanks to a large amount of water/ice molecules trapped in the grooves of the surface, while the C8 had the weakest, as depicted in Supporting Information Figure S2. Thus, the C5 surface exhibited the best complementing matching with the ice layer, which is highly likely to lead to strong interlocking if the graphene surface was positionally fixed.

**Figure 3:**
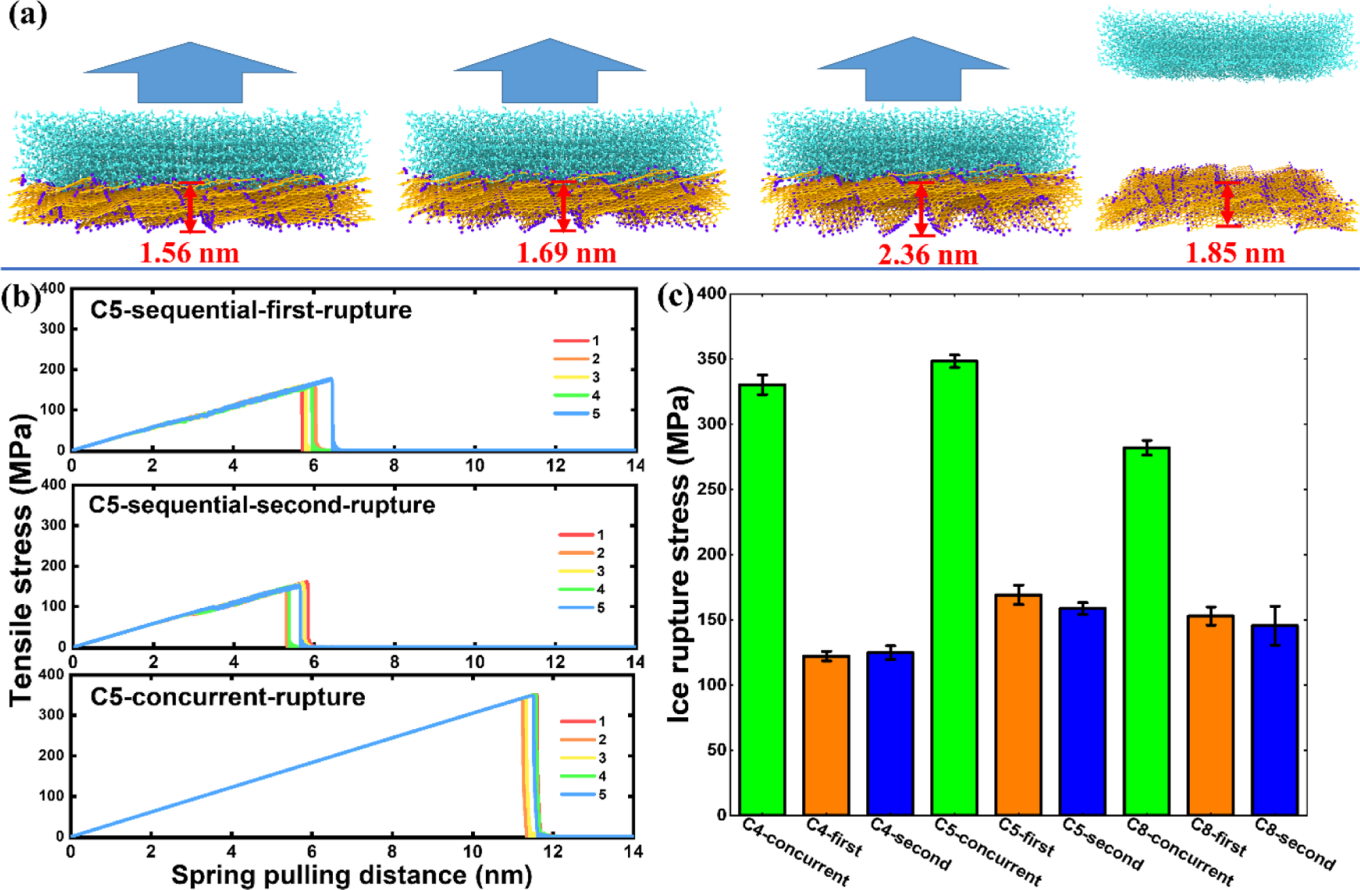
Deicing testing on the fish-scale-like surfaces. (a) Representative system snapshots in the sequential rupture process of cyclic tensile detaching from the C5 surface. The pulling force is indicated by the arrow. The changes in the thickness of the surfaces are highlighted by the red bar. (b) Pulling stress profiles in cyclic deicing tests on the C5 surface. The sudden drop in the pulling stress represents ice detaching events in each independent simulation. Pulling stress responses obtained in the concurrent rupture mode on the C5 surface are plotted for comparison (bottom). (c) Ice rupture stress observed on the three surfaces, including cyclic sequential and concurrent rupture stress. The error bars denoted standard deviations of five independent runs.

The atomistic ice adhesion strength of the three fish-scale-like surfaces was first compared in a cyclic deicing test in order to verify the effect of sequential rupture. To do so, the tensile detaching process of the ice layer from the surfaces was carried out using the same procedure as in the former studies.^19,31,36^ As the representative ice detaching event given in Supporting Information Movie (pulling-process.mp4), the ice layer was first slowly lifted from the surface under the increasing pulling force and finally detached from the substrate, as shown in Figure 3a. All the tensile stress on the ice layer featured a steady increase owing to the constant pulling speed of the harmonic spring and the slow movement of the ice, as shown in Figure 3b. Because the pulling force was applied on the COM of the ice layer, the counterforce came from the interaction between the ice and the surfaces. When the tensile stress reached the critical value σ_r_, the ice layer was fully detached from the surfaces, resulting in a sudden drop at the end of the tensile stress curve. Under the concurrent and sequential rupture modes, the fish-scale-like surfaces reacted to external pulling stress in remarkably different manners. Because all the graphene platelets were fixed, the three surfaces showed no structural change throughout the deicing process in concurrent rupture. In contrast, the average thickness of the graphene platelet layer in the sequential rupture mode first increased due to the opening of the graphene platelets and then decreased after ice detachment, as indicated also in Figure 3a. The concurrent rupture modeled to strong ice adhesion, with tensile ice adhesion strength σ of 330.2 ± 7.5, 348.3 ± 4.8, and 281.9 ± 5.6 MPa for the C4, the C5, and the C8 surfaces, respectively. The difference in the concurrent σ on the three surfaces was correlated with the combined effects of the local structures of the ice–surface interface and the atomistic interactions (Supporting Information Figures S2 and S3). Better accommodation of water/ice molecules in the surface roughness grooves and the resulting stronger interfacial interaction between the ice and the surface have resulted in higher ice adhesion strength, with C5 showing the strongest ice–surface interaction and ice adhesion. Strikingly, the sequential rupture mode in cyclic deicing tests on the three surfaces resulted in around a 50% reduction in σ_r_ as shown in Figure 3c, which was 122.2 ± 3.7, 169.1 ± 7.4, and 152.9 ± 6.9 MPa for the C4, C5, and C8 surfaces, respectively. The result of a significant decrease in σ_r_ further confirmed the effect of the sequential rupture in lowering ice adhesion.^19^ The σ_r_ by the sequential rupture mode obtained in cyclic deicing tests on each surface was stable, as demonstrated in Figure 3c by the similar σ_r_ values monitored in the first and second rounds of deicing on each of the three surfaces. Although the local structure of the ice–substrate interface had changed after the first detaching event and the subsequent re-adhesion of ice (see below), the σ_r_ by sequential rupture was not significantly affected. The key determinant of σ_r_ by sequential rupture was the rupture mode of detachment rather than the local structure of the ice–substrate interface.

**Figure 4:**
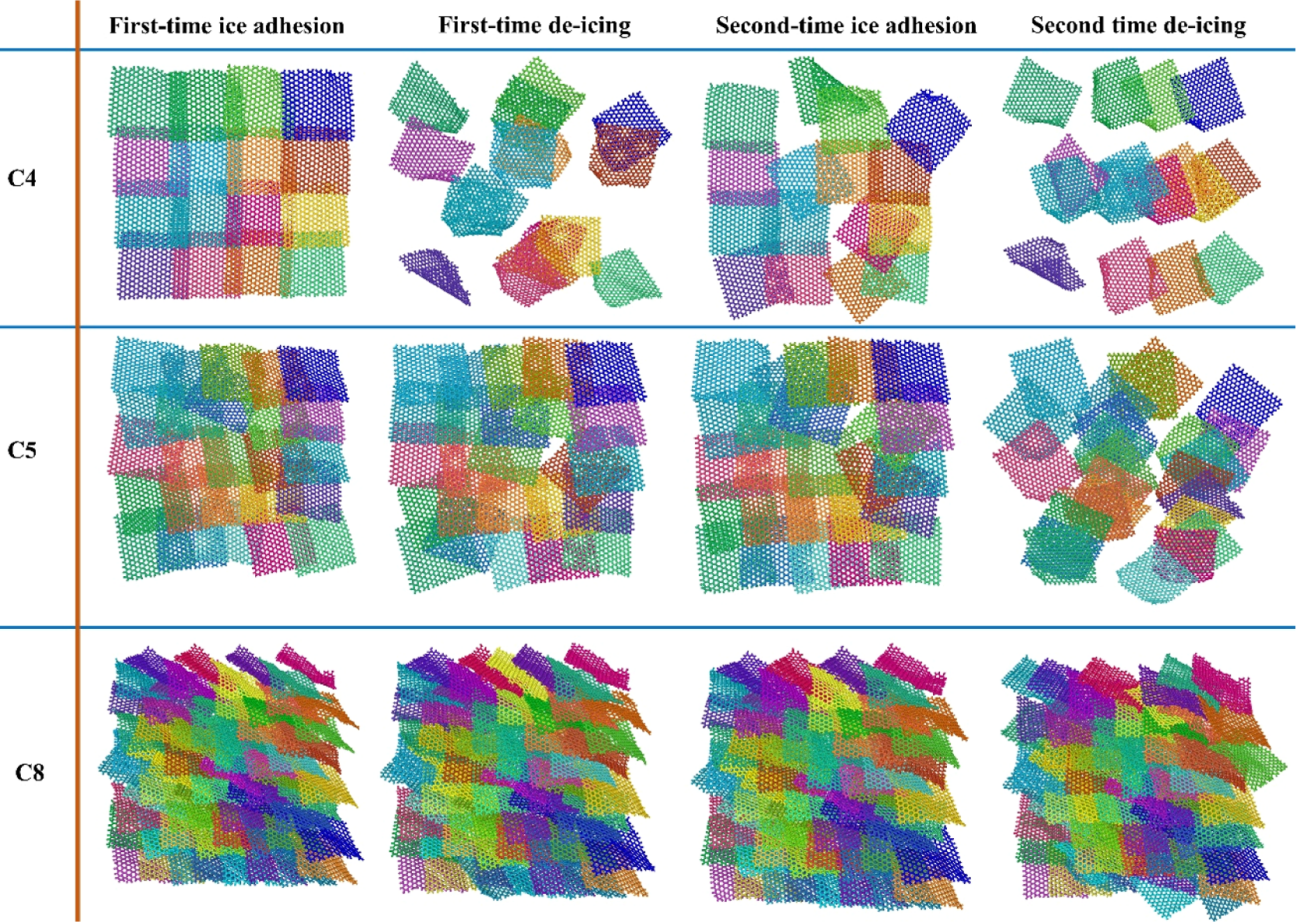
Top view of the three fish-scale-like surfaces in the cyclic deicing tests. The packing morphologies of the three surfaces, C4, C5, and C8, after the first equilibrated ice adhesion, first-time deicing, second-time ice adhesion, and second-time deicing, were shown in subsequence horizontally. Each graphene platelet on the surfaces is colored differently for better visualization.

The reversibility of the hierarchical morphologies on biological surfaces such as gecko toes and water strider legs is crucial for the reproducibility of their special surface mechanical properties.^24,25^ For these natural surfaces, the arrangement of the surface units, especially the pattern and packing density, is key to the surface durability. It is critical for these natural surfaces to resist any mechanical damage and, in the worst scenario, to recover rapidly from damage. Mimicking the abilities of damage resistance is an important aim of the fish-scale-like surfaces. The reversibility of the hierarchical structure of the three fish-scale-like surfaces in the cyclic deicing tests was put together for comparison, as shown in Figure 4. Specifically, the C8 surfaces demonstrated excellent reversibility of graphene platelet orientation and surface coverage after the cyclic deicing tests. Thanks to the close packing of the graphene platelets, the top half of the graphene platelets responded to the ice adhesion and detachment events, while the lower half of the graphene platelets maintained a close-packed structure not affected by the deicing forces. At the end of the deicing tests, the graphene platelets showed a re-adjusted position and yet similar morphology as before the deicing test. Importantly, the C8 gave full coverage of the *XZ*-plane, which indicated an ability to produce a sequential rupture mode. In contrast, the surface morphology of C5 and C4 was significantly altered in the cyclic deicing test. The C5 surface was already partially damaged after the first round of deicing, as shown in Figure 4. Although the fish-scale-like structure of C5 was slightly restored in the ice re-adhesion equilibration simulation, the final arrangement of the graphene platelets was completely distorted if compared to the original state. The surface area of C5 was not fully covered by the graphene platelets after the second round of deicing, which was an indication of breakages. The C4 showed the least reversibility in morphology. Not only was the fish-scale-like structure lost but also a large area of open space was not covered by the graphene platelets (top row in Figure 4). Given that the three surfaces consist only of the graphene platelets, the open space without coverage thus becomes the contact area between the ice and the solid substrate below the graphene platelets in reality. Such areas can serve as large interlocking points for enhanced ice adhesion,^41,42^ which can greatly weaken the anti-icing properties of the surfaces. Here, the open areas did contribute to the low ice adhesion strength in the second round of the deicing test observed on the C4 and C5 because there were no atomistic interactions between the ice and the surface. The low ice adhesion resulting from the sequential rupture on the C4 and C5 surfaces thus was not sustainable, given the surface structures easily destroyed by external forces.

**Figure 5:**
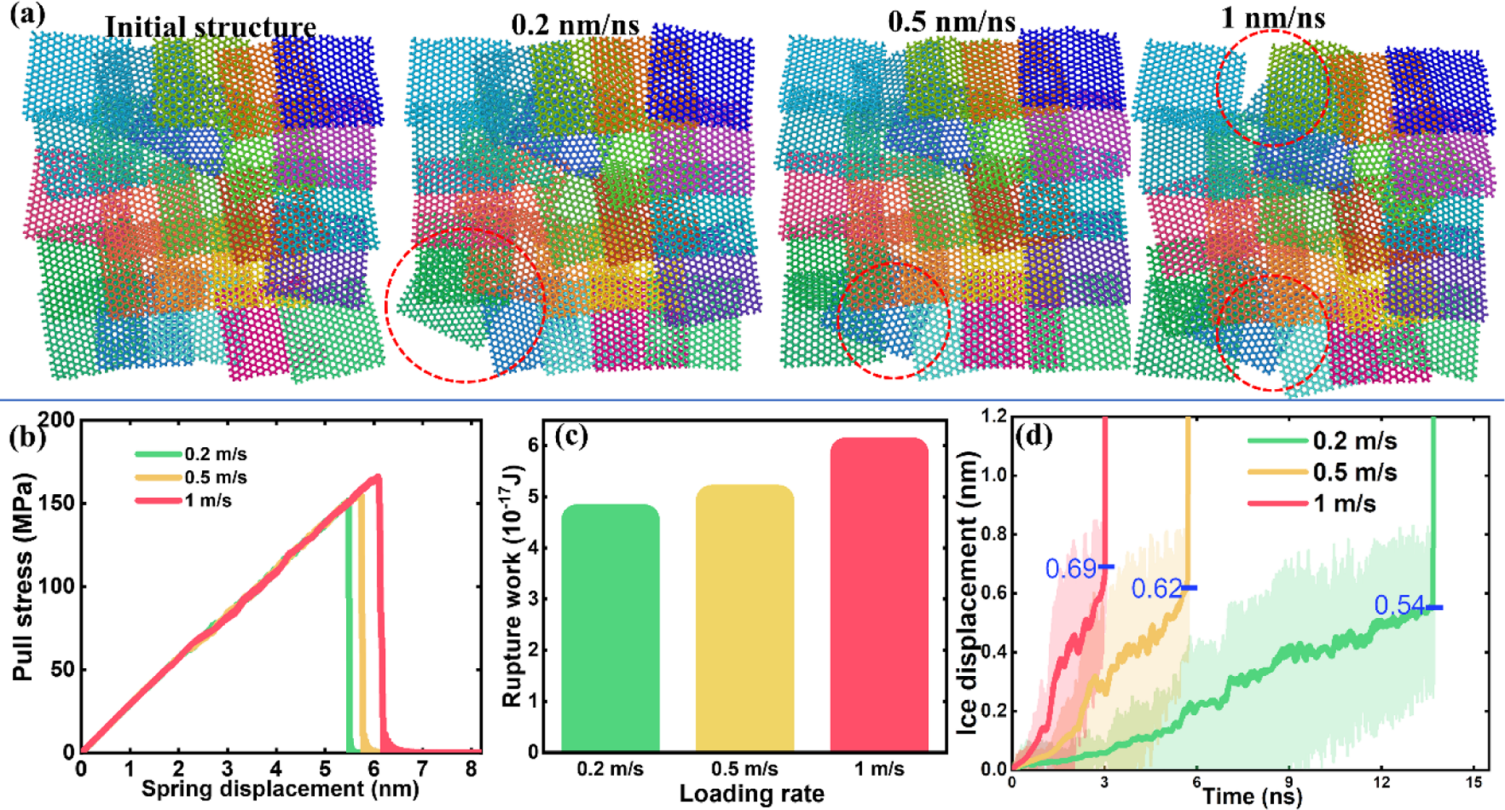
Deicing on the C5 surface with different pulling rates. (a) Surface morphologies of the C5 before and after deicing tests with different pulling rates. Random areas not covered by graphene platelets after deicing are highlighted by red dashed circles. The corresponding pulling rate of each resulting morphology is highlighted in the figure. (b) Typical pulling stress profiles observed in the deicing with varied pulling rates. (c) Loading-dependent rupture work of the ice from the C5 surface. (d) Ice displacement under pulling force with corresponding pulling speed given. The displacement of ice where the detaching event happened is marked in blue.

It is known that a higher loading rate, determined by the spring tensile pulling speed, can lead to higher rupture stress.^19,31^ The loading rate thus influences the deicing behavior and morphology reversibility of the surfaces. For comparison, two extra tensile pulling speeds of 0.2 and 1 nm/ns were chosen for the deicing test on the C5 surface. As the surface snapshots after first-round deicing are shown in Figure 5a, the morphologies showed no obvious differences after deicing with the three tensile pulling speeds. The main characteristic of the fish-scale-like arrangement of the graphene platelets remained after the ice detachment event, with random areas not covered by graphene platelets. Because the pulling rates tested here ranged only 1 order of magnitude, the resulting rupture stress monitored was 161.7, 164.6, and 171.0 MPa for the loading rates of 0.2, 0.5, and 1 nm/ns, respectively, as shown in Figure 5b. The difference in the resulting rupture stress was less than 10 MPa. As it is known that rupture stress increases logarithmically with the loading rate,^43^ a much higher pulling loading rate is needed to generate an obvious difference in rupture stress, which is beyond the scope of this work. The increase in rupture stress was ∼6% from the lowest to the highest pulling rate (from 161.74 to 170.96 MPa). In accordance with the rupture stress, the rupture work needed for complete deicing also increased with the pulling rate, as depicted in Figure 5c. Surprisingly, the rupture work showed an increase of ∼25% with the increasing pulling rate from 0.2 to 1 nm/ns, in contrast to the slight increase in the rupture stress. The obvious increase in rupture work indicated the surface had varied sequential opening distances under different tensile pulling rates. Indeed, as shown by the ice displacement distances before the rupturing event in Figure 5d, a lower tensile pulling rate led to lower ice upward movement and thus the sequential opening distance of the surface. Because the sequential opening distance is correlated with the displacement of the graphene platelets from their original position, a higher pulling rate can result in large displacement and thus an increased probability of distortion of the graphene platelets and damage to the surfaces.

### 3.2 Shearing Ice on the Fish-Scale-Like Surfaces

**Figure 6:**
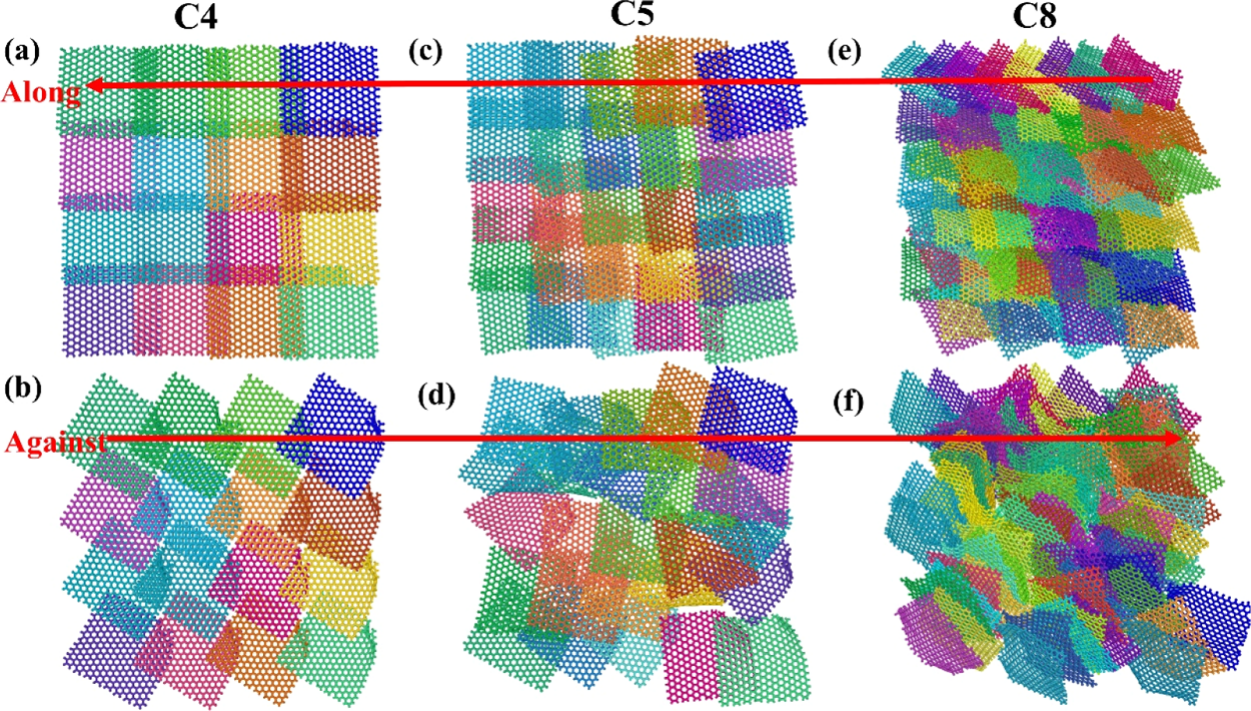
Top view of the morphology of graphene platelets on the three surfaces after ice shearing along and against the ordered direction of the platelet in the sequential mode, with (a,b) for the C4, (c,d) for the C5, and (e,f) for the C8, respectively. Here, the red arrow indicates the direction of ice motion.

Because of the anisotropic structure of the fish-scale-like surfaces, shear stress was expected to exhibit different features along and against the graphene platelet orientation direction.^19^ Furthermore, the different packing densities of graphene platelets on the three surfaces led to significant variation in the local structure of the ice surface and interaction potential, as shown in Supporting Information Figures S2 and S3, which also contributed to the differences in the shearing of the ice on the three surfaces. For a detailed comparison, ice was sheared on all the three surfaces along and against the graphene platelet orientation, under both the concurrent mode and sequential mode. In contrast to the no structural changes of the surfaces under the concurrent mode, the morphology of graphene platelets can be opened and overthrown in a different direction by external shearing force in the sequential rupture mode. As shown in Figure 6, all the three surfaces in the sequential rupture mode can be easily altered by shearing stress against the ordered direction of the graphene platelets due to the flexibility of the graphene platelets. As such, the fish-scale-like organization of the graphene platelets was maintained in shearing, which was important for deicing. As shown by the shearing movie in Supporting Information Movie (shearing-process.mp4), the sequential rupture mode was able to keep the hierarchical structure of the surfaces.

**Figure 7:**
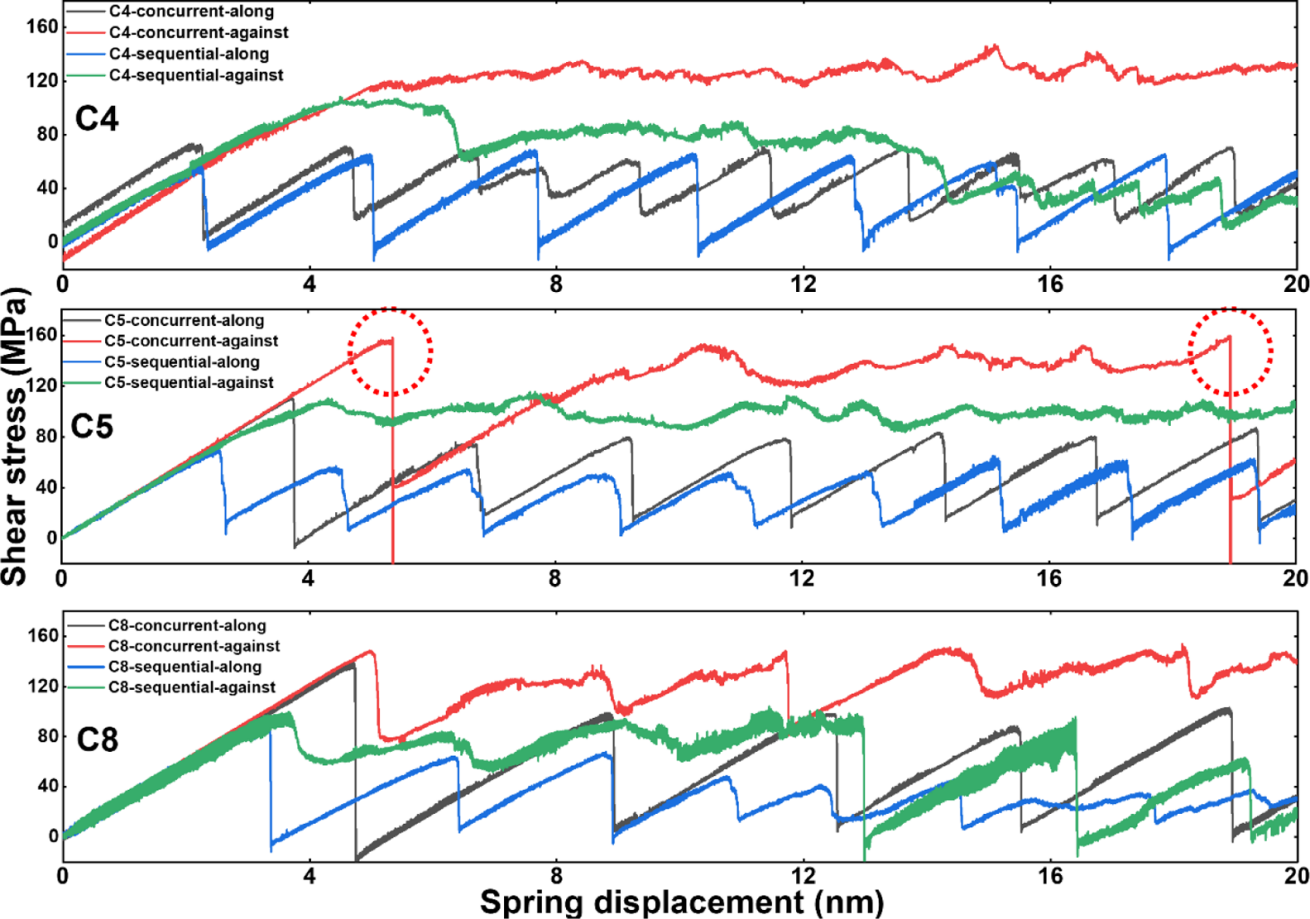
Ice shearing stress profiles on the fish-scale-like surfaces. Shearing stress labeled with “concurrent” legends were obtained with all the graphene platelets fixed, and those labeled with “sequential” legends were obtained with the sequential rupture mode enabled. All the stress profiles shared the same color code in the three plots. Abnormal drops in shearing stress monitored on the C5 surface were highlighted in red dashed circles.

The anisotropic surfaces, namely the uniform arrangement of the graphene platelet orientation, resulted in anisotropy in the observed ice shear stress. The patterns of ice shear stress profiles were similar to the results reported in the previous study, as shown in Figure 7.^19^ Specifically, high shear stress values were observed if the ice was sheared against the graphene platelet orientation, owing to the intercalating ice adhering interface with the fish-scale-like surface. Otherwise, the shear stress was relatively low with saw-teeth-like fluctuations if the ice was sheared along with the graphene platelet orientation. The saw-teeth-like stress pattern was caused by the ice attaching/detaching at the fish-scale-like surface. Because the equilibrated ice adhesion led to the matching of the ice with the periodic low and high repeated surface topography, low shear resistance along the graphene platelet orientation facilitated sliding of the whole ice layer and repeated re-matching between the ice and the surface. The peak value of the shear stress was in the range of 60–80 MPa, as depicted in Figure 7. For all the surfaces, the highest stress was monitored during shearing against the ordered direction of the graphene platelet and with all the platelets fixed in position (the concurrent rupture mode). The peak stress value was close to 140 MPa for all the three surfaces, despite the differences in the platelet packing density. Because the flexibility and re-orientation of the graphene platelets in the sequential rupture mode could relax stress in shearing against the platelet orientation, the corresponding shearing stress decreased gradually in the shearing test. In contrast, constant high shearing stress was observed in ice shearing against the platelet orientation direction in the concurrent mode on all the three surfaces.

**Figure 8:**
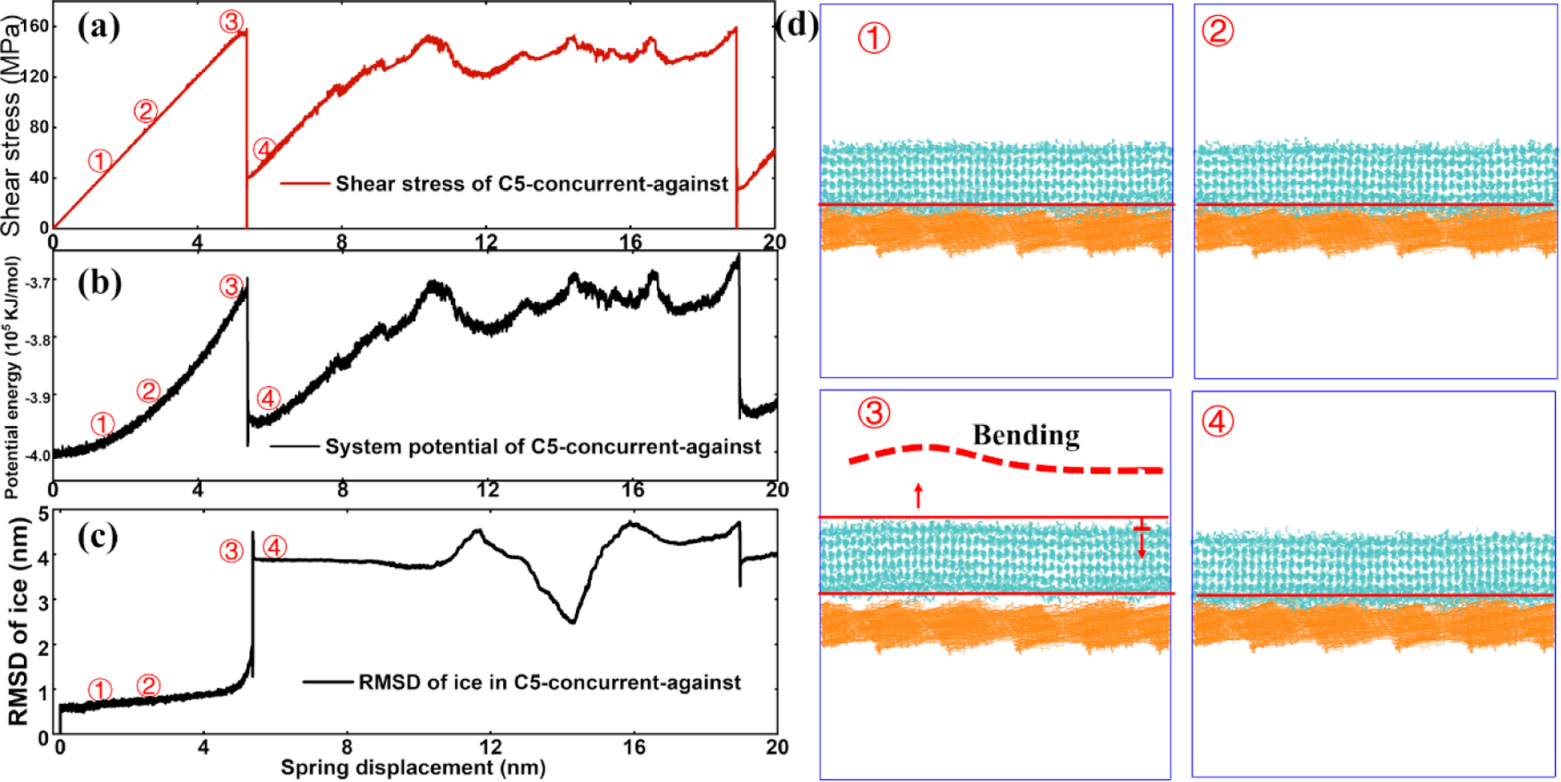
Interlocking breakthrough on the C5 surface. (a) Shear stress profile with abnormal drops observed in shearing ice against the graphene platelet orientation direction, as taken from Figure 7. (b) Corresponding system interaction potential profile in the shearing process. (c) rmsd of the ice layer structure in the whole shear test. (d) System snapshots in an interlocking breakthrough event. The circle number of each snapshot indicated its corresponding position in the stress, potential, and rmsd profile showed in (a–c). The bending of the ice layer was further sketched for better visualization effect.

Given that the graphene platelets of all the three surfaces were fixed in the concurrent mode, the interface matching of the ice and the surfaces became strong interlocking against shearing, especially on the C5 surface. As highlighted in Figure 7, abnormal drops in shear stress were observed in shearing against the platelet orientation, which indicated abrupt changes in the ice structure under high shearing stress. As shown in Figure 8, the observed abnormal drops indeed indicated breakthroughs in the interlocking between the ice layer and the surface. Namely, with the building up of shearing stress, the interaction potential of the system also steadily increased, as shown in Figure 8b. Because the surface structure was fixed in the concurrent mode, the increase in the system potential can be attributed to the changes in the ice structure. The structural root-mean-squared deviation (rmsd) of the ice structure agreed with such an assumption, as shown in Figure 8c. The ice layer buckled under the shearing, as shown in Figure 8d. Under the highest peak of the shear stress, the interlocking between the ice and the surface was destroyed, where the whole ice layer took off from the surface and re-adhered back to the surface in a short time of several picoseconds. The process is captured by system snapshots shown in Figure 8d. The abrupt structure change in the ice layer in interlocking breakthrough events can be expected in ice shearing tests on hard surfaces in experiments.

## 4 Conclusions

In summary, three fish-scale-like surfaces were built by assembling graphene platelets in a uniform ordered orientation. By using cyclic tensile and shearing deicing tests, surfaces with a high packing density of graphene platelets exhibited stable and reversible surface morphology for the reproducibility of sequential ice rupture and the subsequent low atomistic ice adhesion strength. Despite varied ice–substrate interactions resulting from different graphene platelet packing densities, all the surfaces showed a 50% reduction in ice adhesion strength. The low tensile ice adhesion strength on different fish-scale-like surfaces was in a similar range, which signified that the sequential rupture mode was the dominating factor for the reduction of ice adhesion. The high packing density of graphene platelets was key to the full and reversible coverage of the surface, which remained the integrality of the effective structure for deicing. Furthermore, the high packing density was crucial for maintaining a uniform graphene platelet orientation under shearing stress. Motivated by natural surface examples, this work supplied a low intrinsic ice adhesion surface design strategy of deicing, which further verified the sequential rupture as an effective approach for lowering atomistic ice adhesion and at the same time shed light on new icephobic materials that are responsive to external stimuli.

## Supporting Information Available

Pulling process (MP4)
Shearing process (MP4)
Relationship between the surface area and the packing of platelets, roughness landscape of the three fish-scale-like surfaces and the corresponding equilibrated ice adhere interfaces, and interaction potential between the ice layer and the substrate after equilibration adhesion of 100 ns (PDF)
