# Supplementary material for: Assembly of Graphene Platelets for Bioinspired, Stimuli-Responsive, Low Ice Adhesion Surfaces
Source: ACS Omega. 2022 Mar 17;7(12):10225–34. doi: 10.1021/acsomega.1c06782 (PMC8973109; doi:10.1021/acsomega.1c06782)
Supplement: Supplementary file 3 — ao1c06782_si_003.pdf [file ao1c06782_si_003.pdf]

Supporting Information

Assembly graphene platelets for bioinspired, stimuli-responsive, low ice adhesion surfaces

Yuequn Fu[1], Senbo Xiao[1*], Bjørn Helge Skallerud[1], Zhiliang Zhang[1] and Jianying He[1*]

[1]NTNU Nanomechanical Lab, Department of Structural Engineering, Norwegian University of Science and Technology (NTNU), Trondheim 7491, Norway

Supplementary Figures

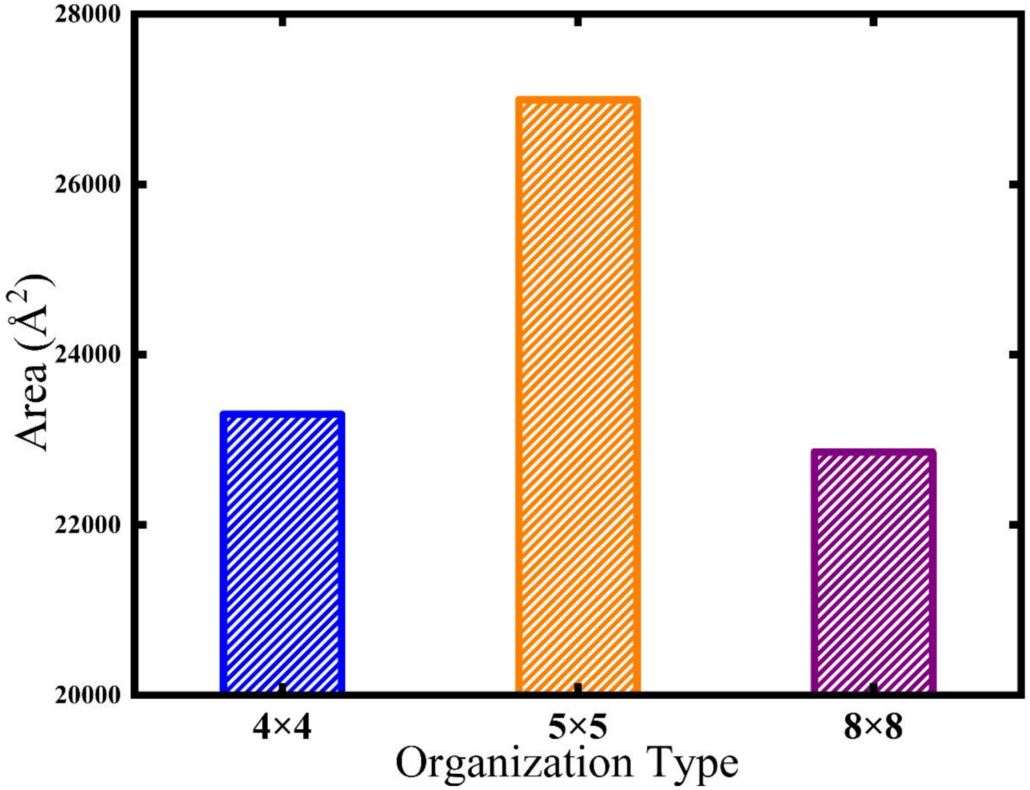

Figure S1. Relationship between surface area and the packing of platelets.

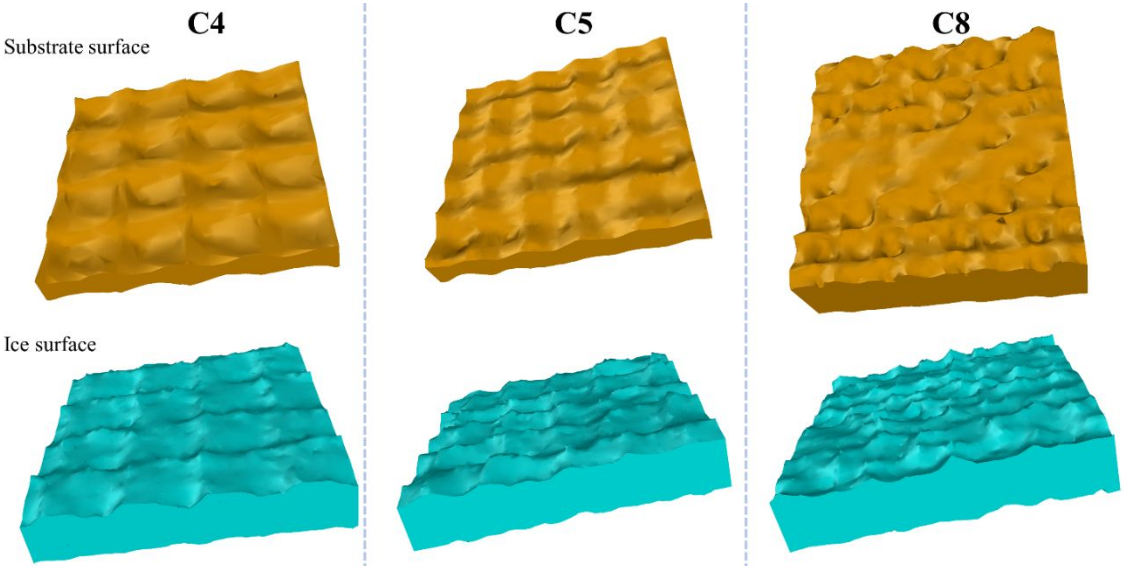

Figure S2. Roughness landscape of the three fish-scale-like surfaces and the corresponding equilibrated ice adhere interfaces.

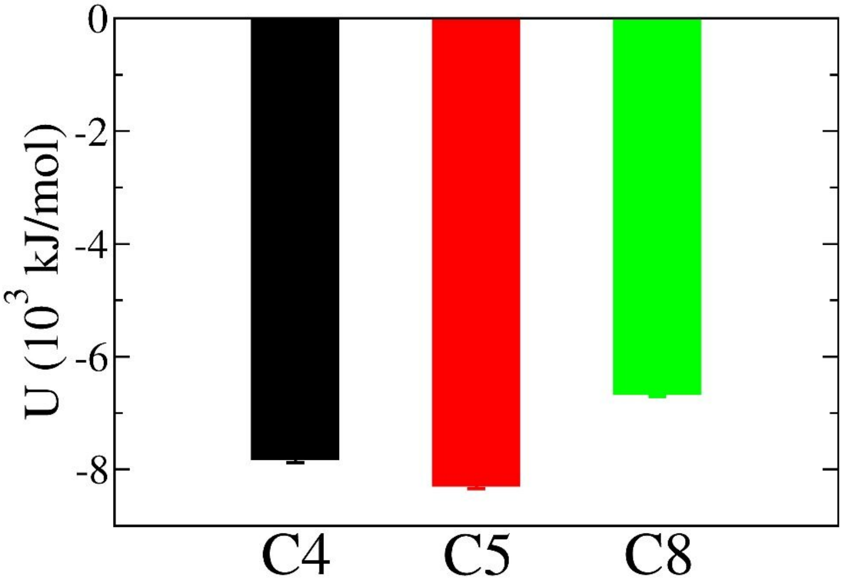

Figure S3. The interaction potential between the ice layer and the substrate after equilibration adhesion of 100 ns. Standard deviation in the potential was showed as error bar.
